# GPI-anchor and GPI-anchored protein expression in PMM2-CDG patients

**DOI:** 10.1186/1750-1172-8-170

**Published:** 2013-10-20

**Authors:** Maria E de la Morena-Barrio, Trinidad Hernández-Caselles, Javier Corral, Roberto García-López, Irene Martínez-Martínez, Belen Pérez-Dueñas, Carmen Altisent, Teresa Sevivas, Soren R Kristensen, Encarna Guillén-Navarro, Antonia Miñano, Vicente Vicente, Jaak Jaeken, Maria L Lozano

**Affiliations:** 1 Centro Regional de Hemodonación Servicio de Hematología y Oncología Médica. Hospital Universitario Morales Meseguer, Universidad de Murcia, Ronda de Garay S/N, 30003 Murcia, Spain; 2 Departamento de Bioquímica, Biología Molecular B e Inmunología, Universidad de Murcia, Murcia, Spain; 3 Departamento de Neurología Infantil, Hospital Sant Joan de Déu, Barcelona, Spain; 4 Unidad de Hemofilia, Hospital Universitario Vall d’Hebron, Barcelona, Spain; 5 Hematology Department, Centro Hospitalar de Coimbra, Coimbra, Portugal; 6 Hematology Department, Aalborg Hospital, Aalborg, Denmark; 7 Unidad de Genética Médica. Servicio de Pediatría, Hospital Clínico Universitario Virgen de la Arrixaca, El Palmar, Murcia, Spain; 8 Center for Metabolic Diseases, Universitair Ziekenhuis Gasthuisberg, Leuven, Belgium

**Keywords:** PMM2-CDG, *N*-glycosylation defects, GPI-anchor and GPI-anchored proteins

## Abstract

**Background:**

Mutations in *PMM2* impair phosphomannomutase-2 activity and cause the most frequent congenital disorder of glycosylation, PMM2-CDG. Mannose-1-phosphate, that is deficient in this disorder, is also implicated in the biosynthesis of glycosylphosphatidyl inositol (GPI) anchors.

**Objective:**

To evaluate whether GPI-anchor and GPI-anchored proteins are defective in PMM2-CDG patients.

**Methods:**

The expression of GPI-anchor and seven GPI-anchored proteins was evaluated by flow cytometry in different cell types from twelve PMM2-CDG patients. Additionally, neutrophil CD16 and plasma hepatic proteins were studied by Western blot. Transferrin glycoforms were evaluated by HPLC.

**Results:**

Patients and controls had similar surface expression of GPI-anchor and most GPI-anchored proteins. Nevertheless, patients displayed a significantly diminished binding of two anti-CD16 antibodies (3G8 and KD1) to neutrophils and also of anti-CD14 (61D3) to monocytes. Interestingly, CD16 immunostaining and asialotransferrin levels significantly correlated with patients’ age. Analysis by flow cytometry of CD14 with MΦP9, and CD16 expression in neutrophils by Western blot using H-80 ruled out deficiencies of these antigens.

**Conclusions:**

*PMM2* mutations do not impair GPI-anchor or GPI-anchored protein expression. However, the glycosylation anomalies caused by *PMM2* mutations might affect the immunoreactivity of monoclonal antibodies and lead to incorrect conclusions about the expression of different proteins, including GPI-anchored proteins. Neutrophils and monocytes are sensitive to *PMM2* mutations, leading to abnormal glycosylation in immune receptors, which might potentially affect their affinity to their ligands, and contribute to infection. This study also confirms less severe hypoglycosylation defects in older PMM2-CDG patients.

## Introduction

**Figure 1:**
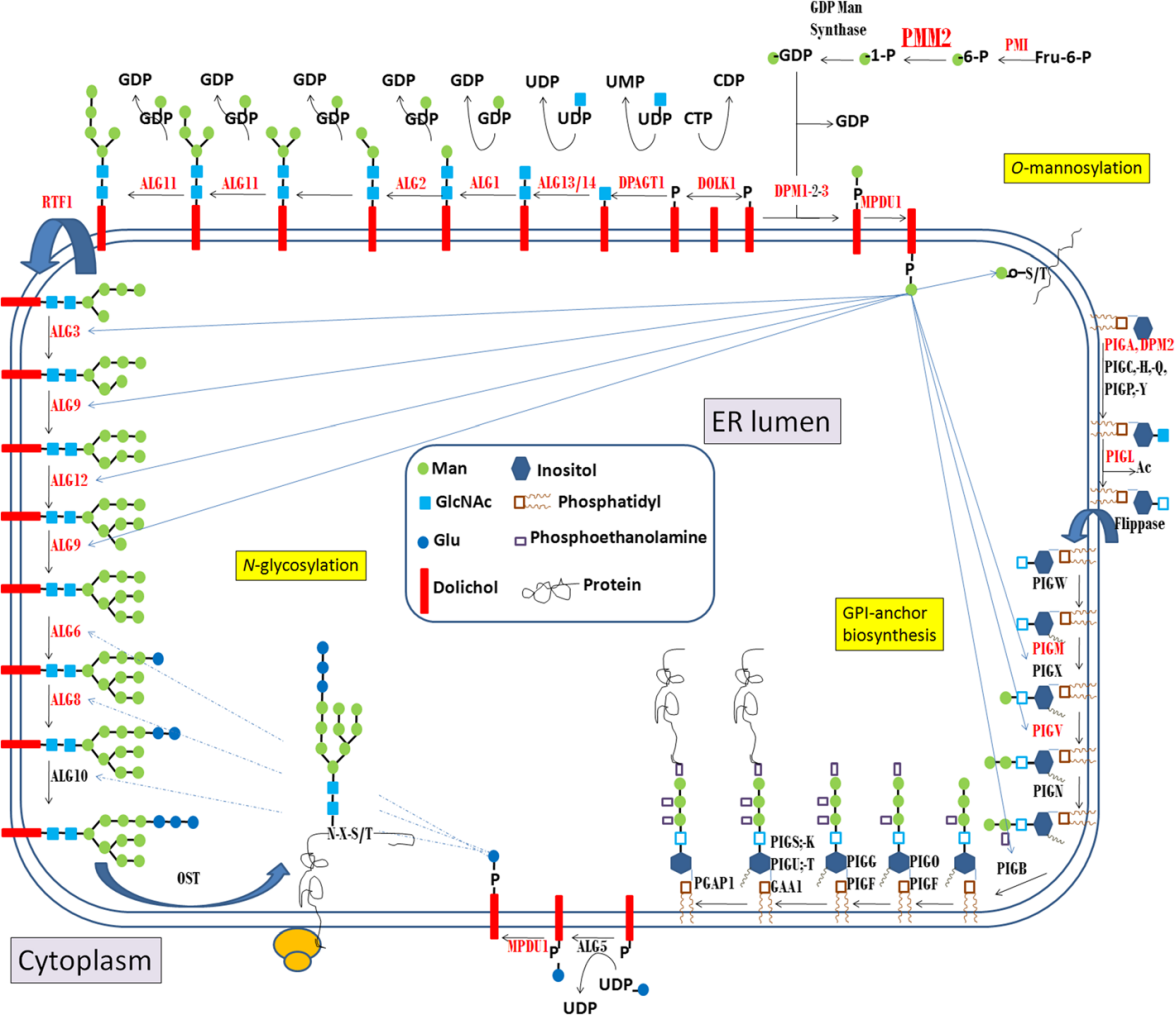
**Biosynthetic pathways depending on mannose-1-phosphate: *N*-glycosylation, GPI-anchor biosynthesis, and *O*-mannosylation.** Mannose-1-phosphate is the product of the phosphomannomutase activity, encoded by the *PMM2* gene. The steps involved in the synthesis of the lipid-linked oligosaccharides required for the *N*-glycosylation of proteins, and glycosylphosphatidyl inositol required for the anchor of other proteins are detailed. The genes involved in each step are indicated, and those with mutations associated with congenital disorders of glycosylation are marked in red.

Congenital disorders of glycosylation (CDG) are caused by defective glycosylation of glycoproteins and glycolipids. Up to date more than 70 CDG have been reported [1-3]. These disorders show an extremely broad clinical spectrum involving nearly all organs, with degrees of severity that range from early death to very mildly affected adults [4]. The number of inborn defects of glycosylation is expected to further increase rapidly [1]. The most prevalent *N*-linked disorder, CDG-Ia, first reported in 1980, is an autosomal recessive defect caused by deficient phosphomannomutase (PMM) activity [5] due to mutations in *PMM2*[6]. Thus, this disorder is now called PMM2-CDG [7]. PMM2 catalyzes the conversion of mannose-6 phosphate to mannose-1-phosphate, an early step in the assembly of the lipid-linked oligosaccharide (LLO) precursor required for *N*-glycosylation (Figure 1). The most widely used method to screen for *N*-glycan synthesis defects is isoelectric focusing of serum transferrin (Tf), showing a type 1 pattern in PMM2-CDG (increase of asialo- and disialotransferrin) [8].

Two other metabolic pathways also require mannose-1-phosphate, although with lower requirements than *N*-glycosylation (nine mannose residues): glycosylphosphatidyl inositol (GPI) anchor biosynthesis (three mannose residues), and *O*-mannosylation synthesis (one mannose) (Figure 1) [9]. PMM2-CDG patients have no symptoms of α-dystroglycan hypomannosylation. However, the identification of mutations in the genes for the 3 subunits of dolichol-phosphate-mannose (DPM) synthase in patients with *N*-glycosylation defects and muscular dystrophy-dystroglycanopathy syndrome [10] prompt further studies to address whether impaired phosphomannomutase activity might also affect other metabolic pathways requiring dolichol phosphate-mannose, like *O*-mannosylation. Moreover, as far as we know, there is no study evaluating GPI-anchor and GPI-anchored protein expression in PMM2-CDG patients. Since GPI-anchored proteins are involved in multiple pathways, defects in these proteins may contribute to the complex phenotype of this disease. For example, the tendency towards thrombosis in patients with PMM2-CDG could be multifactorial, and potentially (at least in part), due to deficiency of GPI-anchored complement inhibitors on the surface of circulating erythrocytes and other cells [11]. In this context, it has to be noted that two GPI-anchor synthesis defects, paroxysmal nocturnal haemoglobinuria (PNH), and PIGM-CDG (Figure 1), exhibit a high rate of occurrence of severe thrombotic events due to complement-mediated hemolysis resulting from the deficiency of complement regulatory proteins, CD55 and CD59 [12,13].

This study aimed to evaluate the expression of GPI-anchor and GPI-anchored proteins in different cell types from PMM2-CDG patients.

## Material and methods

### PMM2-CDG patients and controls

Twelve PMM2-CDG patients from different European countries were enrolled in this study. Venous blood was collected into citrate-tubes and delivered within 24–48 hours by express courier at room temperature to Murcia, Spain, where all further studies were done.

At least one healthy control subject was recruited for each patient, and samples were drawn and shipped under the same conditions as described above. A total of 25 healthy controls, including 5 children, were also enrolled in this study.

Controls, patients, and relatives were fully informed of the aim of this study, which was performed according to the declaration of Helsinki, as amended in Edinburgh in 2000, and they gave written informed consent. This study obtained approval from the Reina Sofia Hospital Ethics Committee.

### Analysis of abnormal glycoforms of hepatic proteins, antithrombin levels and PMM2 molecular study

A complete diagnostic workup of PMM2-CDG patients was performed by looking for protein *N*-glycosylation defects in three different hepatic proteins: transferrin, antithrombin, and α1-antitrypsin. Transferrin glycoforms were evaluated by HPLC as previously described [14]. Briefly, plasma samples were iron saturated, lipid precipitated and filtrated. Glycoforms were separated by HPLC, and detected at 470 nm. To assess for antithrombin defects, we determined anti-FXa activity using a chromogenic method, and the presence of abnormal glycoforms in plasma was evaluated by Western blot essentially as reported [15]. Hypoglycosylated forms of α1-antitrypsin were detected by Western blot using a polyclonal antibody (Dako, Glostrup, Denmark). Molecular analysis of all 8 exons and flanking regions of the *PMM2* gene was performed as previously described [16].

### Flow cytometry analysis

Flow cytometry is the method of choice for identifying cells deficient in GPI-anchor linked proteins. In this study we followed the guidelines for the diagnosis and monitoring of a disease caused by such a deficiency, PNH, recently published by the International Clinical Cytometry Society (ICCS) [17] and by the Spanish Society of Haematology and Haemotherapy [18]. Briefly, for red blood cells we performed a no lyse-no wash procedure; for FLAER white blood cells were lysed-washed and then stained, while for white blood cells a stain-then-lyse procedure was used. For granulocyte evaluation we analyzed the following combinations of monoclonal antibodies (mAb): CD16-PE, CD45-PerCP, CD64-APC; CD24-PE, CD45-PerCP, CD64-APC; CD55-PE, CD45-PerCP, CD64-APC. For monocyte assessment we used CD14-PE, CD45-PerCP, CD64-APC. For erythrocyte evaluation we performed analysis of CD61-FITC, CD55-PE; CD61-FITC, CD59-PE (Table 1). Additionally, FLAER (fluorochrome-conjugated (Alexa 488) fluorescein-labelled proaerolysin) which binds specifically to the GPI anchor was used to analyze GPI-deficient myeloid and lymphoid populations (together with CD45-PerCP and CD64-APC). Briefly, 50,000-100,000 events were collected using a FACScalibur flow cytometer (Becton Dickinson, Mountain View, CA), and analysis was performed with the Paint-a-Gate software (Becton Dickinson) and CellQuest (Becton Dickinson) to obtain the mean fluorescence intensity (MFI) of stained cells (Additional file 1: Figure S1).

**Table 1:** Cell types and molecules evaluated by flow cytometry in this study

**Cell type**	**Molecule**	**GPI-anchor**	**MoAb**	**Source**
**Erythrocytes**	CD55	Yes	1A10 (PE)	Becton-Dickinson
	CD59	Yes	MEM-43 (PE)	Caltag
**Monocytes**	CD14	Yes	MФP9 (PE)	Becton-Dickinson
			61D3(FITC)	eBioscience
	CD55	Yes	1A10 (PE)	Becton-Dickinson
	CD48	Yes	J4-57	Beckman-Coulter
	CD33	No	WM53 (PE)	BD Pharmingen
	FLAER	GPI	--(Alexa 488)	Pinewood Scientific
**Lymphocytes**	CD48	Yes	J4-57	Beckman-Coulter
	CD3	No	SK7 (FITC)	Becton-Dickinson
	CD19	No	HIB19(PE-Cy5)	BD Pharmingen
	CD16	No	3G8 (PE)	BD Pharmingen
	FLAER	GPI	--(Alexa 488)	Pinewood Scientific
**Neutrophils**	CD16	Yes	KD1	Culture supernatant
			3G8 (PE)	BD Pharmingen
			3G8 (PE-Cy5)	BD Pharmingen
	CD55	Yes	1A10 (PE)	Becton-Dickinson
	CD24	Yes	ML5 (PE)	BD Pharmingen
	CD66	Yes	Kat4c	Dako
	CD54	No	HA58 (PE)	BD Pharmingen
	FLAER	GPI	--(Alexa 488)	Pinewood Scientific

**MoAb**: Monoclonal antibody.

### Erythrocyte analysis

We evaluated potential qualitative rather than quantitative defects on the erythrocyte GPI-linked complement inhibitor, CD59, which might contribute to the thrombotic phenotype of PMM2-CDG patients. For this purpose, we assayed A) whether the red blood cells (RBCs) in patients were lysed by complement when normal serum was acidified by the acidified-serum lysis test (Ham test), and B) if reduced ionic strength of serum by addition of an iso-osmotic solution of sucrose could activate the classic complement pathway, and complement-sensitive cells would then be lysed by the sucrose test [19,20].

### Localization of epitopes recognized by anti-CD16 and anti-CD14 antibodies

To analyze the epitope recognition by KD1 and 3G8 pair of mAb on CD16 antigen, and by 61D3 and MФP9 mAb on CD14 antigen binding, competition experiments were performed. Briefly, whole blood cells from healthy donors were incubated first with the unconjugated KD1 mAb, washed and then stained with the second conjugated mAb (3G8-PE-Cy5). We also performed CD14 immunostaining by incubation of cells with FITC-conjugated 61D3, and after a washing step, they were stained with PE conjugated MФP9- mAb. The fluorescence intensity was analyzed by flow cytometry, and compared to that displayed by direct staining with the fluorochrome-conjugated 3G8- PE-Cy5 or MФP9-PE mAb on neutrophils or monocytes, respectively [21].

### Neutrophil isolation and CD16 evaluation by Western blot

Neutrophil isolation and Western blot analysis were done essentially as previously described [22]. Briefly, after dextran sedimentation to remove red blood cells, we performed a Ficoll-Hypaque sedimentation to separate mononuclear cells from neutrophils. The neutrophil pellet was removed and a hypotonic lysis step was done to eliminate the remaining erythrocytes. Neutrophils (3.75×10^5^) were lysed with 10x volume ice-cold lysis buffer [10 mM TrisHCl, 0.5 mM DTT, 0.035% SDS, 1mM EGTA, 50 mM sodium fluoride, 50 μM sodium orthovanadate, 5 mM benzamidine and 20 mM phenylmethylsulphonyl fluoride (PMSF)], denaturized and loaded in 8% SDS polyacrylamide gels. After separation by electrophoresis, neutrophil proteins were transblotted to PVDF membranes. CD16 steady-state level was evaluated by Western blot using the H-80 polyclonal antibody (Santa Cruz Biotechnology Inc, Dallas, Texas, USA). As loading control, we evaluated the expression of tubulin by Western blot and Ponceau Red staining of the membrane.

### Statistical analysis

%MFI values are described as mean ± SD. Study groups were compared by Mann–Whitney U test. P-values <0.05 were considered statistically significant.

Correlations between both CD16 and asialotransferrin values according to age were analyzed by linear regression using SPSS software. P-values <0.05 were considered statistically significant.

## Results

The characteristics of the PMM2-CDG patients included in this study are shown in Table 2.

**Table 2:** Clinical, demographic, laboratory and genetic characteristics of PMM2-CDG patients

**Patient**	**Country**	**Age (years)**	**Sex**	***PMM2 *** **mutations**	*Transferrin		**AT	**Clinical severity**
					**Asialo**	**Disialo**		
**P1**	Spain	1	M	Y64C, R141H	11.0	45.8	46	Severe
**P2**	Spain	14	M	R141H, E93A	10.0	32.8	35	Moderate
**P3**	Spain	6	F	P113L, T118S+P184D	23.6	44.1	22	Severe
**P4**	Spain	1	M	E33X,V44A	21.5	38.2	17	Severe
**P5**	Spain	4	F	P113L haploinsuficiency	13.5	33.9	53	Severe
**P6**	Belgium	13	M	F119L, R141H	6.0	30.1	43	Moderate
**P7**	Belgium	1	M	P113L, T237R	27.6	40.5	17	Moderate
**P8**	Belgium	8	M	D188G, V231M	9.4	36.1	42	Moderate
**P9**	Belgium	35	F	P113L, R141H	12.7	35.7	28	Mild
**P10**	Belgium	35	F	P113L, R141H	8.7	32.6	34	Mild
**P11**	Portugal	18	M	R141H, C241S	8.0	25.0	48	Mild
**P12**	Denmark	12	M	F119L homozygous	11.9	39.1	28	Moderate
**Control Pool**	-	-	-	-	1.3	0.8	100	-

*Area of asialo- and disialo- transferrin HPLC peaks (% of total transferrin).

**AT: anti-FXa activity of antithrombin as % of the value observed in a pool of 100 healthy blood donors.

### GPI-anchor and GPI-anchored protein expression

**Figure 2:**
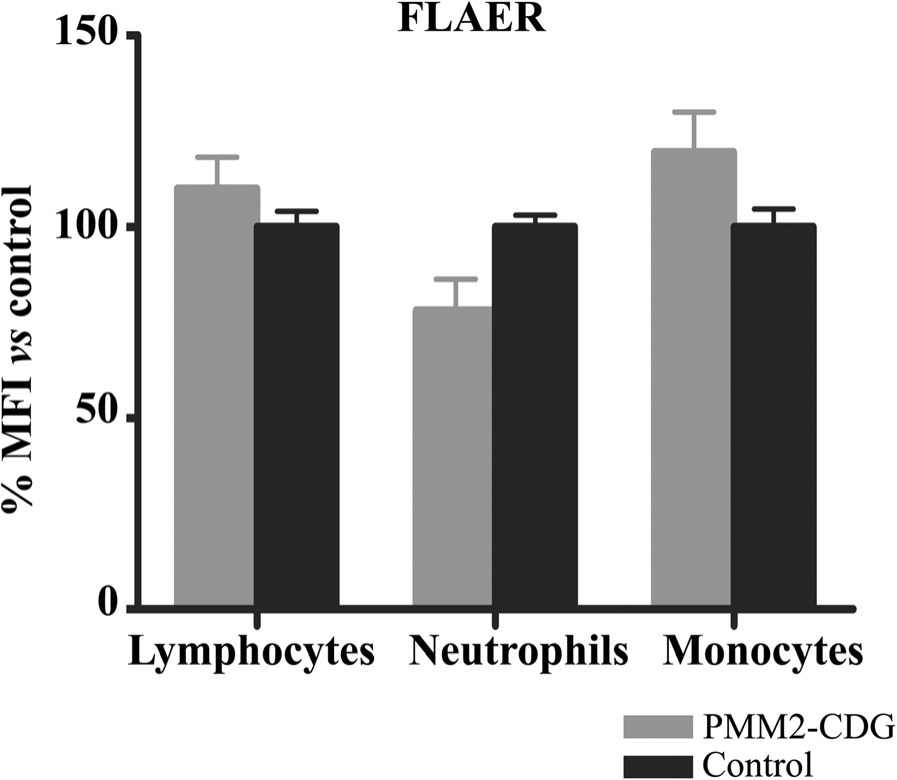
**Level of expression of glycosylphosphatidyl inositol (GPI) anchored-proteins on different blood cells in PMM2-CDG patients and control subjects.** The study was done by flow cytometry using proaerolysin variant (FLAER). Values are expressed as % mean fluorescence intensity (MFI) *vs* that observed in controls. PMN: Polymorphonuclear cells.

**Figure 3:**
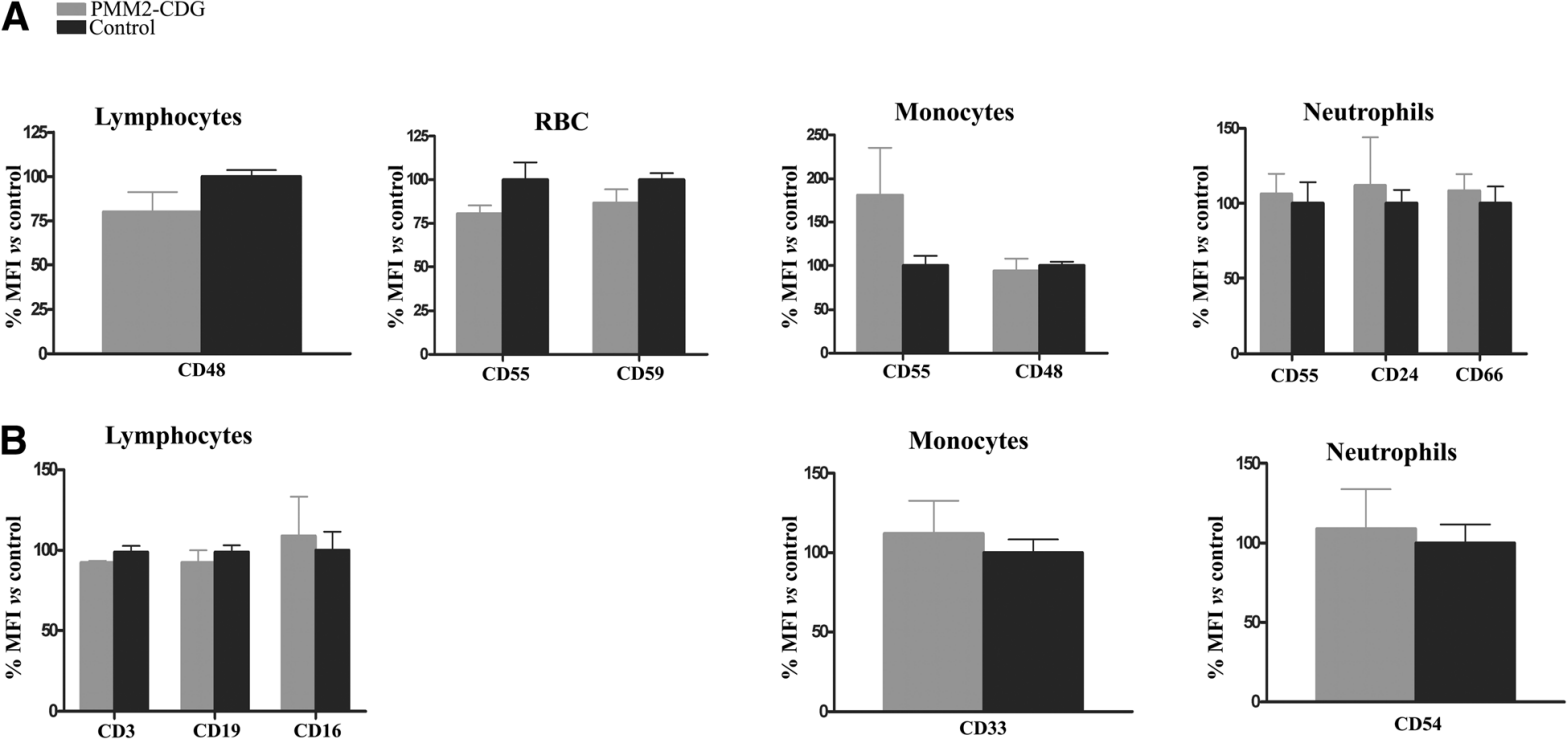
**Expression of A) GPI-anchored proteins, and B) non GPI-anchored proteins on different blood cells in PMM2-CDG and control subjects.** The study was done by flow cytometry using the monoclonal antibodies indicated in Material and Methods. Values are expressed as % mean fluorescence intensity (MFI) *vs* that observed in controls. RBC: red blood cells.

We found that the level of FLAER binding to white blood cells from patients was similar to that of healthy individuals (Figure 2). In agreement with this result, a similar surface expression of most specific GPI-anchored proteins was observed in PMM2-CDG patients and in controls on lymphocytes (CD48), erythrocytes (CD55, and CD59), monocytes (CD55) and neutrophils (CD55, CD24 and CD66) (Figure 3A). A similar expression of non-GPI anchored proteins was also observed in patients and in controls on lymphocytes (CD3, CD19, and CD16), monocytes (CD33) and neutrophils (CD54) (Figure 3B).

**Figure 4:**
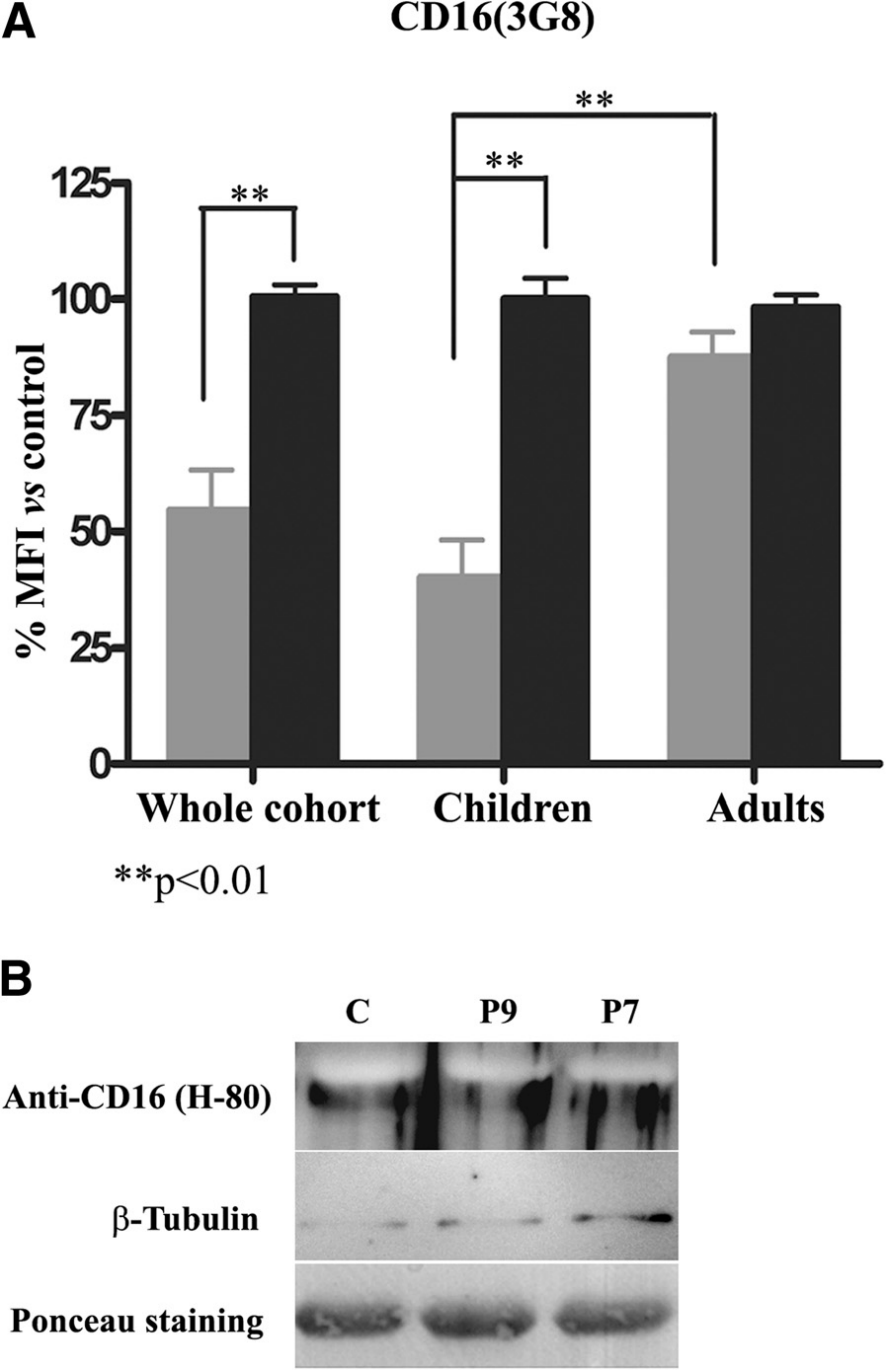
**Expression of CD16 in neutrophils of PMM2-CDG patients and control subjects. A)** Flow cytometry analysis using 3G8 monoclonal antibody in the whole cohort of patients and according to age. Values are expressed as % mean fluorescence intensity (MFI) *vs* that observed in controls. **B)** Western blot analysis using the H-80 polyclonal antibody. As loading controls, we stained the membrane with Ponceau Red and evaluated the expression of tubulin.

However, analysis of CD16 on granulocytes, performed in parallel by two different laboratories (Centro Regional de Hemodonación and Departamento de Bioquímica, Biología Molecular B de Inmunología), revealed that PMM2-CDG patients displayed a significantly diminished CD16 immunostaining using 3G8 mAb compared to controls (Figure 4A). In contrast, the staining of CD16 in lymphocytes, where this molecule exists as a transmembrane non GPI-linked form, was similar in PMM2-CDG patients and controls (Figure 3B). Two patients, P4 and P8, showed negligible binding of anti-CD16 using two different mAb: 3G8 and KD1 (Table 3). Interestingly, these two mAb seem to recognize different but close epitopes since preincubation with KD1 clone strongly reduced 3G8 staining of PMN cells (Additional file 2: Figure S2A). The reduction of CD16 expression on neutrophils seemed to be restricted to children with PMM2-CDG, since the three available adult PMM2-CDG patients displayed normal values (Figure 4A). To analyze whether the decreased CD16 immunostaining was a real deficiency of the molecule, or a defect in recognition by the antibody, we performed Western blot analysis using a polyclonal antibody (H-80) against neutrophils. This assay revealed similar levels of CD16 in neutrophils in PMM2-CDG and in controls (Figure 4B).

**Table 3:** Expression of surface proteins in P4 and P8 PMM2-CDG patients evaluated by flow cytometry

	**Neutrophils**					**Lymphocytes**
	**CD16**		**CD55**	**CD24**	**CD66**	**CD16**
	**3G8**	**KD1**	**1A10**	**ML5**	**Kat4c**	**3G8**
**P4**	5.3%	4.5%	125.7%	123.7%	71.6%	126.8%
**P8**	10.8%	6.4%	56.8%	57.3%	ND	196.2%

Values are expressed as % mean fluorescence intensity *vs* that observed in parallel controls.

ND: not determined.

**Figure 5:**
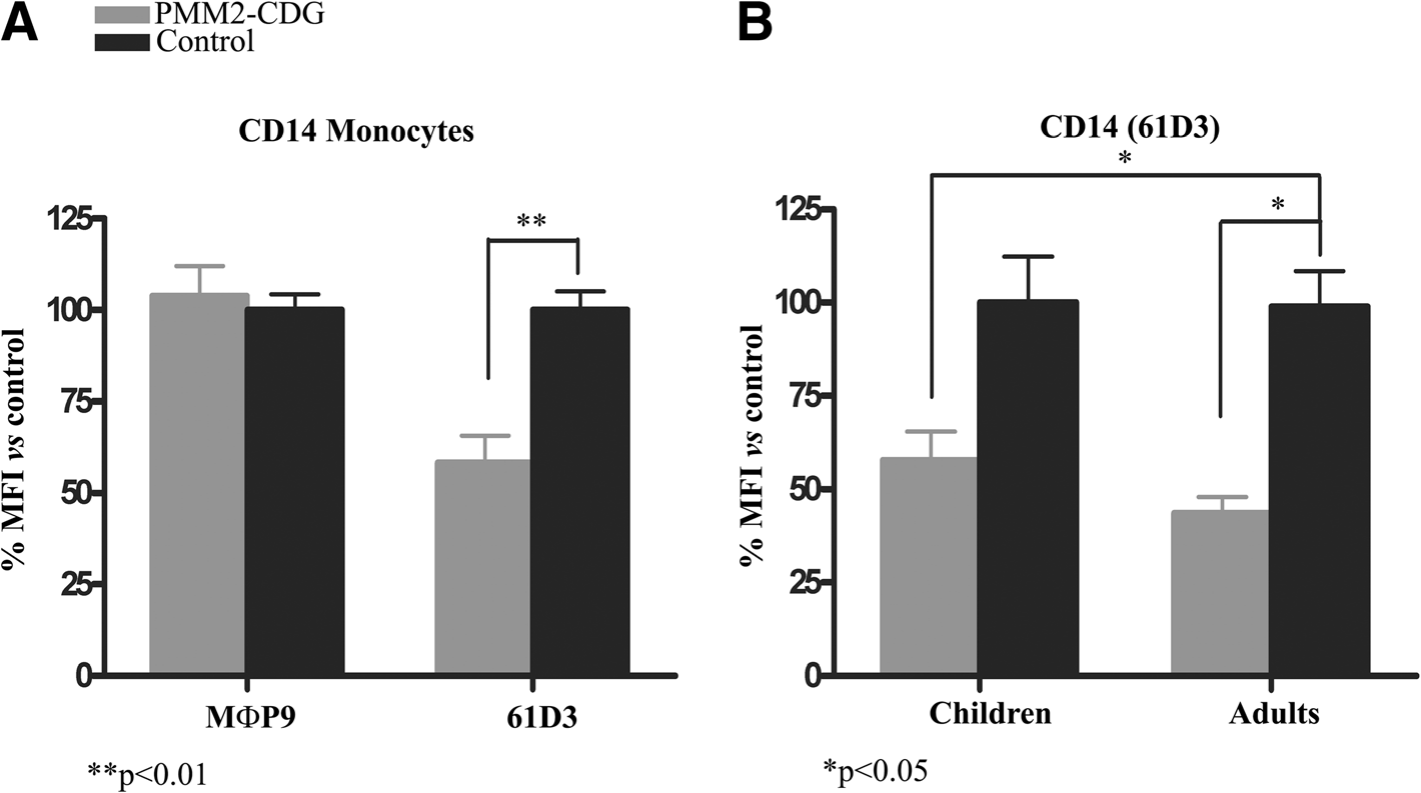
**Expression of CD14 in monocytes of PMM2-CDG patients and control subjects evaluated by flow cytometry. A)** Results obtained with MФp9 and 61D3 monoclonal antibodies in the whole cohort of patients. **B)** Expression of CD14 in monocytes according to age. The study was done with the 61D3 monoclonal antibody. Values are expressed as % mean fluorescence intensity (MFI) *vs* that observed in controls. **p< 0.01; *p< 0.05.

It is also worth to note that opposite results were observed in CD14 staining on monocytes depending on the monoclonal antibody used in flow cytometry analysis. MФP9 revealed similar staining of CD14 in PMM2-CDG patients and in controls (p= 0.400) (Figure 5A). In contrast, 61D3 consistently identified a significant decrease in CD14 binding on PMM2-CDG monocytes (p= 0.002) (Figure 5A). These antibodies seem to recognize distant epitopes since preincubation of monocytes with one anti-CD14 antibody does not affect the staining ability of the second one (Additional file 2: Figure S2). Contrary to what was seen with the specific reduction in CD16 granulocyte expression in children, the decrease of anti-CD14 61D3 binding to monocytes was observed both in children and adults with PMM2-CDG (Figure 5B).

### Erythrocyte lysis

The Ham’s acidified-serum and the sucrose tests were negative in all PMM2-CDG patients, indicating no significant qualitative defect on the erythrocyte surface leading to increased sensitivity to cell lysis mediated by complement (data not shown).

### N-glycosylation analysis of hepatic proteins

**Figure 6:**
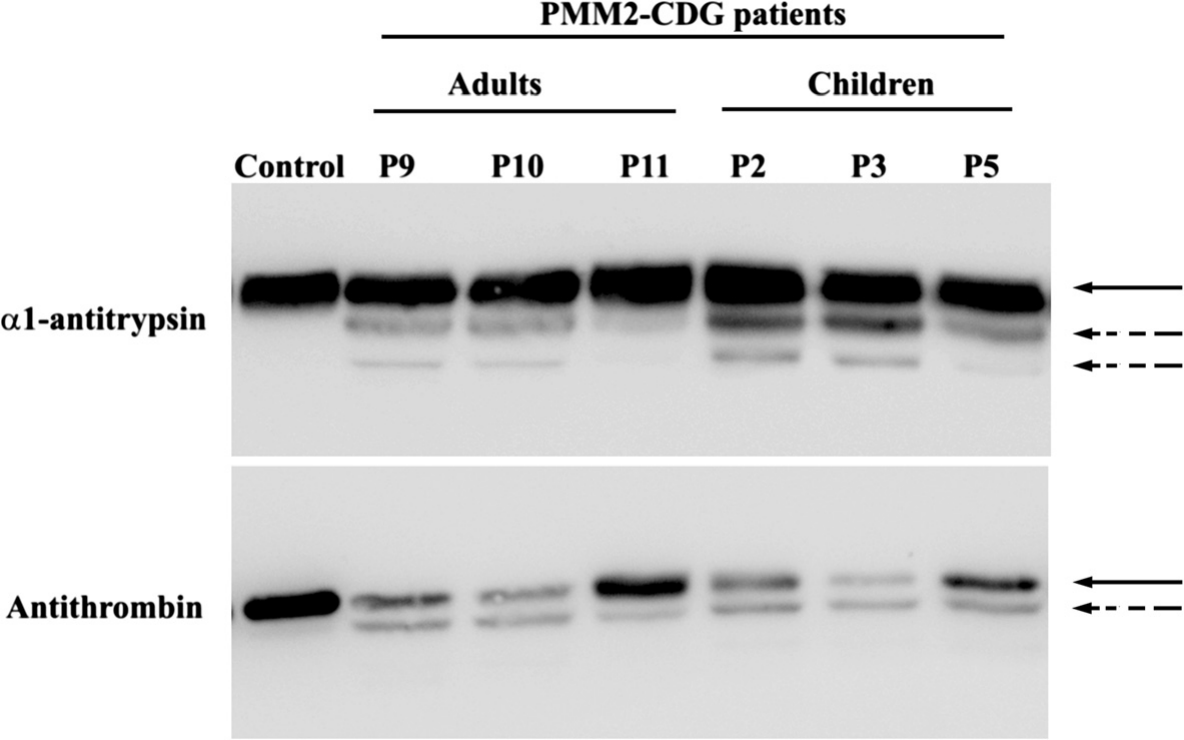
**Glycoforms of antithrombin and α1**-**antitrypsin in plasma of one control, three adults, and three children with PMM2-CDG.** Normal glycoforms are indicated by solid arrows and hypoglycosylated glycoforms by dashed arrows.

All patients exhibited a characteristic HPLC pattern for PMM2-CDG, with increased asialo- and disialotransferrin fractions (Table 2). Similarly, all patients had significant antithrombin deficiency (Table 2) and increased levels of abnormal glycoforms of antithrombin and α-antitrypsin (Figure 6).

## Discussion

Since mannose-1-phosphate is required for the synthesis of both LLO and GPI-anchor, one would suspect that patients with congenital defects affecting enzymes involved in the early and common steps of these pathways might have defects in both the glycan content of *N-*glycoproteins and the GPI-anchor/GPI-anchored protein expression (Figure 1). Actually, GPI-anchored protein deficiencies are included among the congenital disorders of glycosylation [1,12,22-24]. However, as far as we know, no study has evaluated any potential effect of congenital disorders of glycosylation on GPI-anchor and GPI-anchored protein expression. In this study we have addressed this issue in the most prevalent CDG: PMM2-CDG. We have analyzed a broad range of GPI-anchored proteins in four different blood populations from 12 PMM2-CDG patients with different ages, gender, *PMM2* mutations and clinical expression of disease. Our study suggests that in contrast to the significant effect on *N*-glycosylation, mutations in *PMM2* have negligible effects on GPI-anchor and GPI-anchored protein expression in different blood cells. Several hypotheses can be proposed to explain this observation: i, The synthesis of GPI-anchors requires a smaller number of mannoses (three) than the synthesis of LLO (nine) (Figure 1). Thus, the residual PMM activity of PMM2-CDG patients [13,22] could be sufficient to satisfy the requirements for normal GPI-anchor synthesis. ii, It is possible that when GDP-Man and Dol-P-Man are in limited supply, they are used more efficiently in other pathways, so that GPI anchoring occurs but *N*-glycosylation is incomplete, as suggested by Thomas and coworkers [25]. iii, A different turnover time of these mannosylated structures. iv, Elements with the ability to modulate *PMM2* defects by different mechanisms (PMM1 by redundancy, or PGM and PMI by affecting the balance of complementary pathways) might generate mannose-1-phosphate specifically used in the GPI-anchor synthesis. Further studies are required to validate these hypotheses. Independently of this, our study shows that neither the GPI-anchor nor the GPI-anchored protein expressions are affected in PMM2-CDG patients, with the restriction that we have not tested whether the GPI-anchors of these patients are qualitatively affected or show heterogeneity.

According to these results, quantitative defects of GPI-anchor/GPI-anchored proteins are unlikely to be involved in the multisystemic clinical expression of PMM2-CDG patients. We were particularly interested to investigate whether a potential CD59 defect in erythrocytes of PMM2-CDG patients might contribute to their increased incidence of thrombosis [11,15,26]. This defect is usually evaluated through the complement-mediated lysis of erythrocytes, similar to patients with acquired (PNH) or with congenital GPI deficiency (PIGM-CDG) [27]. The normal expression of CD59 in RBC of PMM2-CDG individuals observed by flow cytometry analysis, the negative results observed in the Ham’s and sucrose tests, and the absence of anemia in PMM2-CDG patients [4] argue against quantitative and qualitative CD59 defects in these patients. Therefore, in PMM2-CDG patients, mechanism(s) different from complement-mediated hemolysis, seem to be involved in the disturbance of the hemostatic system leading to increased risk of thrombosis. This may be associated with the significant deficiencies of relevant anticoagulant elements (antithrombin, protein C and protein S) although they also have deficiencies of procoagulant factors (especially Factor IX and XI) associated with a significant deficiency of relevant anticoagulant elements (antithrombin, protein C and protein S) and procoagulant factors (factor IX and XI) [28].

Additionally, CD16 and CD14 immunostaining of neutrophils and monocytes, respectively, was found to be significantly reduced in PMM2-CDG patients by flow cytometric analysis with 3G8 and KD1 (CD16) and 61D3 (CD14) monoclonal antibodies. CD16, the low affinity Fcγ receptor III for IgG (FcγRIII) exists as a polypeptide-anchored form (FcγRIIIA, or CD16-A) in human natural killer cells and monocyte/macrophages, and as a GPI-anchored form in human neutrophils (FcγRIIIB or CD16-B) [29]. CD14 is a component of the innate immune system that along with the Toll-like receptor 4 and MD-2, acts as a co-receptor for the detection of bacterial lipopolysaccharide and also exists in two forms: either anchored into the membrane by a GPI tail (mCD14), or present in a soluble form (sCD14) [30]. Reduced CD16 expression is likely to contribute to the impaired clearance of immune complexes [31]. Moreover, a CD14 deficient animal model exhibits higher bacterial disease severity [32]. Consequently, the decreased CD16 and CD14 expression could potentially contribute to explain the recurrent infections described in PMM2-CDG patients [33,34]. However, several lines of evidences from our study suggest that these patients express normal levels of CD16 and CD14 in neutrophils and monocytes, respectively. The apparent deficiency detected by 3G8, KD1, and 61D3 can be explained by the ability of these antibodies to recognize an epitope influenced by varying proportions of mannose contents, resulting from the *PMM2* mutations of these patients. Indeed, a previous study has shown that the immunoreactivity of the 3G8 epitope on CD16 expressed on neutrophils is dependent primarily on a high mannose-type oligosaccharide, according to the sensitivity to the endo H digestion of the receptor [35], and KD1 may recognize a very close epitope (Additional file 2: Figure S2). Moreover, it has been shown that the FcγIII receptors of natural killer cells and neutrophils differ in their affinity to IgG because of primary sequence differences between the two receptor isoforms, and also by cell type-specific glycosylation of the receptor [36], which justifies the different CD16 expression on lymphocytes and neutrophils of PMM2-CDG patients. Further studies must investigate if the abnormal glycosylation of CD16 in neutrophils and CD14 in monocytes of PMM2-CDG patients affects the function of these immune receptors, as suggested for CD16 [35], which might potentially be implicated in the recurrent infections of these patients.

**Figure 7:**
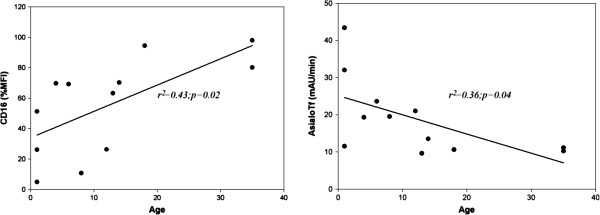
**Correlations of CD16 binding to neutrophils (%mean fluorescence intensity *-*MFI- *vs *controls) and asialotransferrin according to age in PMM2**-**CDG patients.**

The reduced staining of CD16 on neutrophils (detected by 3G8 or KD1 binding) was striking in children, but not in the three adult PMM2-CDG patients analyzed. Interestingly, significant correlations between both CD16 and asialotransferrin with age were observed (Figure 7). These results agree with the suggested stabilization of glycosylation defects with age described in a previous study [37], and might explain the progressive normalization of the transferrin profile in patients who initially present with an abnormal pattern [38], although further studies including serial analysis of additional patients comparing values at different points in time should be performed to fully validate it. Indeed, age-related changes of glycoproteins currently represent an active research area [39]. It remains to be determined whether these changes might also have prognostic relevance in PMM2-CDG patients, as most of the PMM2-CDG patients commonly present with a severe clinical phenotype including recurrent infections, in the first years of life and show a relatively steady clinical picture by puberty [40].

In conclusion, *PMM2* mutations do not impair GPI-anchor or GPI-anchored protein expression. However, the glycosylation anomalies caused by *PMM2* mutations might affect the immunoreactivity of monoclonal antibodies and thus lead to incorrect conclusions about the expression of different proteins, including GPI-anchored proteins. Our results also suggest that neutrophils and monocytes are sensitive to *PMM2* mutations, and relevant immunologic molecules could have abnormal proportion of mannose content with potential functional consequences that might contribute to explain the recurrent infections of PMM2-CDG patients. Finally, our study confirms the stabilization of glycosylation defects with age.

## Competing interests

The authors declare that they have no competing interests.

## Authors’ contributions

MEM-B, TH-C, RG-L, and MLL carried out the flow cytometry studies. MEM-B, IM-M, JC, and AM carried out the HPLC, Western blot and antithrombin studies. MEM-B, JC, AM, and MLL carried out the study of erythrocytes. BP-D, CA, TS, SRK, EG-N, VV and JJ carried out the clinical and genetic studies. TH-C and RG-L carried out the epitope recognition study. MEM-B, TH-C, JC, VV, and MLL carried out the statistical analysis. MEM-B, JC, VV, JJ and MLL designed the study and drafted the manuscript. All authors read and approved the final manuscript.

## Supplementary Material

Additional file 1: Figure S1 Gating strategies used to define granulocytes (blue), monocytes (green), and lymphocytes (red), by (A) forward and side scatter characteristics, (B) immunostaining of CD45, and to better define the monocytic cells, (C) by CD64 expression. Cells within these gates were analyzed independently for expression of GPI and non-GPI molecules by PE-labelled antibodies. The histogram (D) represents CD16 expression in granulocytes from a PMM2-CDG patient (blue) and a control subject (black). PE, phycoerythrin; PerCP, Peridinin chlorophyll protein; APC, allophycocyanin. Click here for file

Additional file 2: Figure S2 Epitope analysis of CD16 and CD14 antibodies. (A) Whole blood cells were either stained directly with PE-Cy5-conjugated 3G8 anti-CD16 mAb (dark-unfilled histogram) or preincubated with unlabelled KD1 anti-CD16 mAb, and then stained with the fluorochrome labelled 3G8 anti-CD16 antibody (light-unfilled histogram) and analyzed by flow cytometry. (B) Whole blood cells were incubated either directly with MФp9 anti-CD14-PE (dark-unfilled histogram) or preincubated first with 61D3 anti-CD14-FITC, and then stained with MФP9 anti-CD14-PE (light-unfilled histogram). Dark-filled histograms represent negative control cells treated with irrelevant isotype-matched control antibody. Click here for file
