# Supplementary figures and images for: GPI-anchor and GPI-anchored protein expression in PMM2-CDG patients
Source: Orphanet J Rare Dis. 2013 Oct 20;8:170. doi: 10.1186/1750-1172-8-170 (PMC4016514; doi:10.1186/1750-1172-8-170)

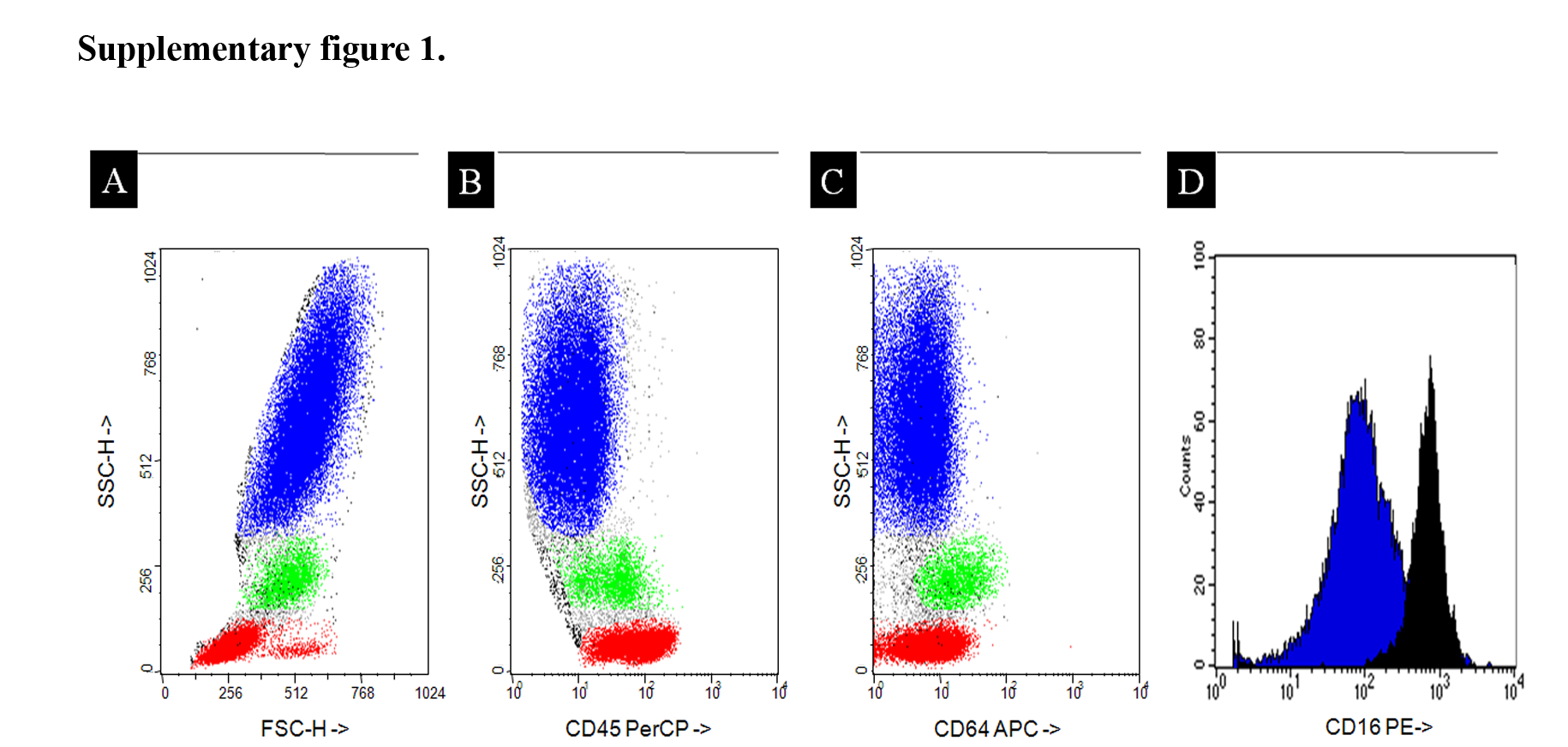

Supplement: Additional file 1: Figure S1 — Gating strategies used to define granulocytes (blue), monocytes (green), and lymphocytes (red), by (A) forward and side scatter characteristics, (B) immunostaining of CD45, and to better define the monocytic cells, (C) by CD64 expression. Cells within these gates were analyzed independently for expression of GPI and non-GPI molecules by PE-labelled antibodies. The histogram (D) represents CD16 expression in granulocytes from a PMM2-CDG patient (blue) and a control subject (black). PE, phycoerythrin; PerCP, Peridinin chlorophyll protein; APC, allophycocyanin. [file 1750-1172-8-170-S1.tiff]

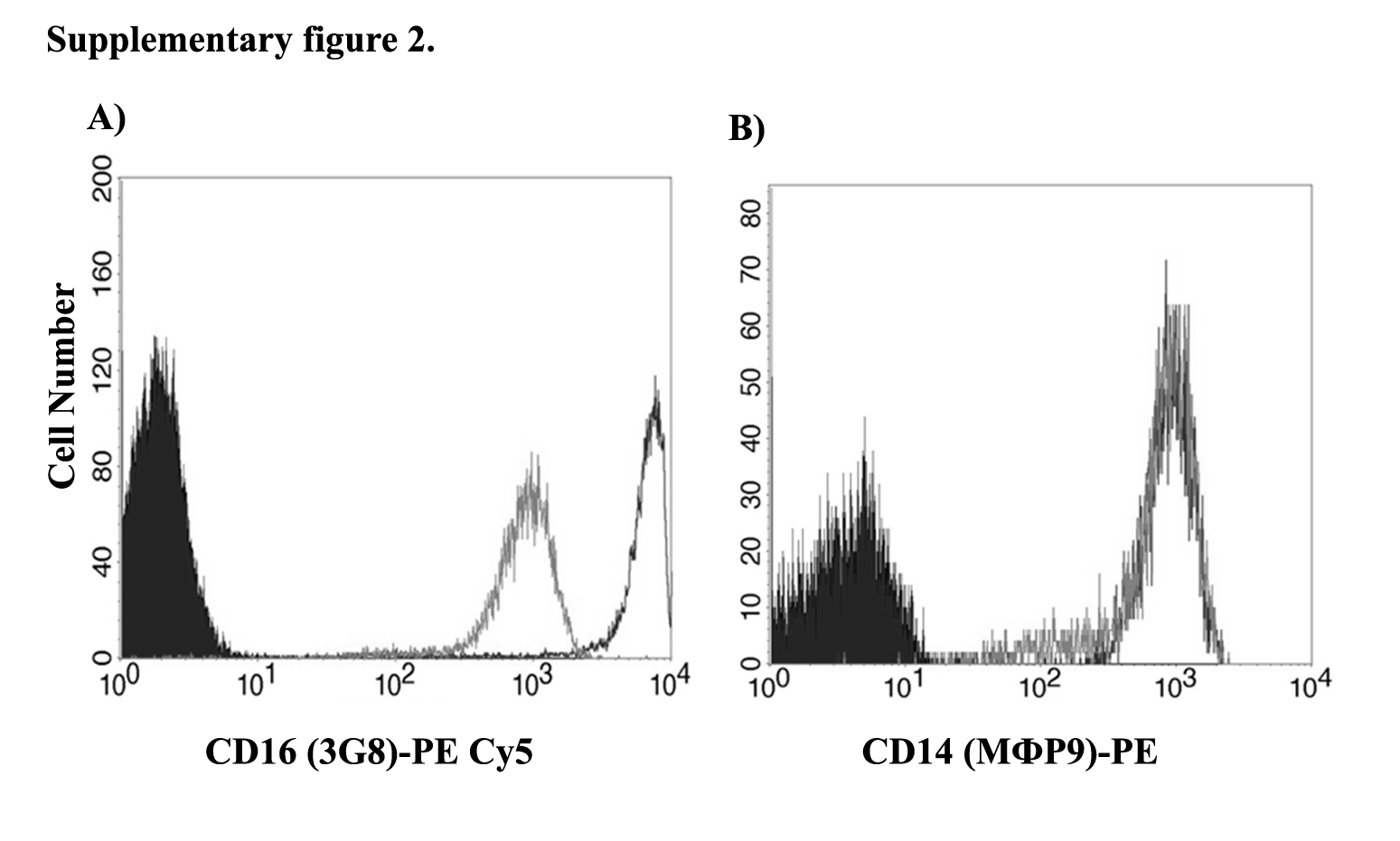

Supplement: Additional file 2: Figure S2 — Epitope analysis of CD16 and CD14 antibodies. (A) Whole blood cells were either stained directly with PE-Cy5-conjugated 3G8 anti-CD16 mAb (dark-unfilled histogram) or preincubated with unlabelled KD1 anti-CD16 mAb, and then stained with the fluorochrome labelled 3G8 anti-CD16 antibody (light-unfilled histogram) and analyzed by flow cytometry. (B) Whole blood cells were incubated either directly with MФp9 anti-CD14-PE (dark-unfilled histogram) or preincubated first with 61D3 anti-CD14-FITC, and then stained with MФP9 anti-CD14-PE (light-unfilled histogram). Dark-filled histograms represent negative control cells treated with irrelevant isotype-matched control antibody. [file 1750-1172-8-170-S2.tiff]
